# Factors associated with non-AIDS-defining cancers and non HCV-liver related cancers in HIV/HCV-coinfected patients- ANRS-CO13 HEPAVIH cohort

**DOI:** 10.1371/journal.pone.0208657

**Published:** 2018-12-18

**Authors:** Oumar Billa, Mathieu Chalouni, Dominique Salmon, Isabelle Poizot-Martin, Camille Gilbert, Christine Katlama, Didier Neau, Julie Chas, Philippe Morlat, Karine Lacombe, Alissa Naqvi, Karl Barange, Anne Gervais, Olivier Bouchaud, Eric Rosenthal, Caroline Lascoux-Combe, Daniel Garipuy, Laurent Alric, Stéphanie Dominguez, Daniel Vittecoq, Cécile Goujard, Claudine Duvivier, Hugues Aumaitre, Patrick Miailhes, David Zucman, Anne Simon, Estibaliz Lazaro, François Raffi, Laure Esterle, Linda Wittkop, Firouzé Bani-Sadr, for the ANRS CO13 HEPAVIH Study Group

**Affiliations:** 1 Univ Bordeaux, ISPED, Inserm Bordeaux Population Health, team MORPH3EUS, UMR 1219, CIC-EC 1401, Bordeaux, France; 2 Université Paris Descartes, Paris, France; 3 Unité Maladies Infectieuses et Tropicales- Hôpitaux Universitaires Paris Centre, APHP, Paris, France; 4 Aix Marseille Université, APHM Hôpital Sainte-Marguerite, Service d’Immuno-hématologie clinique, Marseille, France; 5 INSERM, U912 (SESSTIM)- Marseille, France; 6 Assistance Publique des Hôpitaux de Paris, Hôpital Pitié-Salpétrière, Service Maladies infectieuses et tropicales, Paris, France; 7 Institut Pierre Louis Epidémiologie et Santé Publique UPMC, Sorbonne Université, Paris, France; 8 Centre Hospitalier Universitaire de Bordeaux, Service Maladies infectieuses et tropicales Bordeaux, Hôpital Pellegrin, Bordeaux, France; 9 Université de Bordeaux, Bordeaux, France; 10 Assistance Publique des Hôpitaux de Paris, Hôpital Tenon, Service Maladies infectieuses et tropicales, Paris, France; 11 Centre Hospitalier Universitaire de Bordeaux, Service de médecine interne, Hôpital Saint-André, Bordeaux, France; 12 Assistance Publique des Hôpitaux de Paris, Hôpital Saint-Antoine, Service Maladies infectieuses et tropicales, Paris, France; 13 UMR S1136, Institut Pierre Louis d’Epidémiologie et de Santé Publique, Paris, France; 14 Centre Hospitalier Universitaire de Nice, Service d’Infectiologie, Hôpital Archet 1, Nice, France; 15 Centre Hospitalier Universitaire de Toulouse, Service Gastro-entérologie et hépatologie, Hôpital Purpan, Toulouse, France; 16 Assistance Publique des Hôpitaux de Paris, Hôpital Bichat Claude Bernard, Service des maladies infectieuses et tropicales, Paris, France; 17 Assistance Publique des Hôpitaux de Paris, Hôpital Avicenne, Service Maladies infectieuses et tropicales, Bobigny, France; 18 Université Paris 13 Nord, Bobigny, France; 19 Centre Hospitalier Universitaire de Nice, Service de Médecine Interne et Cancérologie, Hôpital l’Archet, Nice, France; 20 Université de Nice-Sophia Antipolis, Nice, France; 21 Assistance Publique des Hôpitaux de Paris, Hôpital Saint-Louis, Service des Maladies infectieuses et tropicales, Paris, France; 22 Centre Hospitalier Universitaire de Toulouse, Hôpital Purpan, Services des Maladies infectieuses et tropicales, Toulouse, France; 23 Centre Hospitalier Universitaire de Toulouse, Hôpital Purpan, Services de Médecine interne-Pole Digestif, Toulouse, France; 24 UMR 152, IRD, Université Toulouse III, Toulouse, France; 25 Assistance Publique des Hôpitaux de Paris, Hôpital Henri Mondor, Service Immunologie clinique et maladies infectieuses, Immunologie clinique, Créteil, France; 26 Assistance Publique des Hôpitaux de Paris, Hôpital Bicêtre, Hôpitaux universitaires Paris Sud, Service Maladies infectieuses et tropicales, Le Kremlin-Bicêtre, France; 27 Assistance Publique des Hôpitaux de Paris, Hôpital Bicêtre, Hôpitaux universitaires Paris Sud, Service Médecine interne et Immunologie clinique, Le Kremlin-Bicêtre, France; 28 Assistance Publique des Hôpitaux de Paris, Service de Maladies Infectieuses et Tropicales, Hôpital Necker-Enfants malades, Centre d’Infectiologie Necker-Pasteur, IHU Imagine, Paris, France; 29 Centre Hospitalier de Perpignan, Service Maladies infectieuses et tropicales, Perpignan, France; 30 Centre Hospitalier Universitaire de Lyon, Service des Maladies Infectieuses et Tropicales, Hôpital de la Croix Rousse, Lyon, France; 31 Hôpital Foch, unité VIH, Suresnes, France; 32 Assistance Publique des Hôpitaux de Paris, Hôpital Pitié-Salpétrière, Département de Médecine Interne et Immunologie Clinique, Paris, France; 33 Centre Hospitalier Universitaire de Bordeaux, hôpital Haut-Lévèque, Service de Médecine interne et Maladies Infectieuses, Pessac, France; 34 CHU de Nantes, Department of Infectious Diseases, Nantes, France; 35 Université de Nantes, CIC 1413, INSERM, Nantes, France; 36 CHU de Bordeaux, Pôle de Santé publique, Service d’information médicale, Bordeaux, France; 37 Centre Hospitalier Universitaire de Reims, Unité des Maladies Infectieuses et Tropicales, Hôpital Robert Debré, Reims, France; 38 Université Reims Champagne Ardenne, EA-4684 / SFR CAP-SANTE, Reims, France; University of Pittsburgh Centre for Vaccine Research, UNITED STATES

## Abstract

Compared to the general population, HIV-infected patients are at higher risk of developing non-AIDS-defining cancers. Chronic HCV infection has also been associated with a higher risk than that of the general population of developing cancers other than hepatocarcinoma. Evaluation of the impact of HCV-related factors on non-AIDS-defining and non HCV-liver (NANL) related cancers among HIV/HCV co-infected patients are scarce. The aim of this study was to identify the impact of HIV/HCV clinical characteristics on NANL related cancers in a large cohort of HIV/HCV-coinfected patients followed from 2005 to 2017. Cox proportional hazards models with delayed entry were used to estimate factors associated with NANL related cancer. Among 1391 patients followed for a median of 5 years, 60 patients developed NANL related cancers, yielding an incidence rate of 8.9 per 1000 person-years (95% CI, [6.6–11.1]). By final multivariable analysis, after adjustment for sex, tobacco or alcohol consumption, baseline CD4 cell count and HCV sustained viral response (SVR), age and a longer duration since HIV diagnosis were independently associated with a higher risk of NANL related cancer (aHR for each additional year 1.10, 95% CI 1.06–1.14, p<0.0001 and 1.06, 95% CI 1.01–1.11, p = 0.02, respectively). Duration of HCV infection, cirrhosis, HCV viral load, genotype and SVR were not associated with the occurrence of NANL related cancer. Among HIV/HCV-coinfected patients, age and the duration of HIV infection were the only characteristics found to be associated with the occurrence of NANL related cancer. In contrast, no association was observed with any HCV-related variables.

## Introduction

HIV-infected patients have a higher risk of developing non-AIDS-defining cancers compared to the general population [1–4]. Age, duration of HIV infection and immune status are known risk factors for many of these non-AIDS cancers [2,5,6]. Additional factors may vary by cancer, such as poor immune control for oncogenic viruses such as Human papillomavirus (HPV) or Epstein Barr virus (EBV), and/or lifestyle choices, such as tobacco or alcohol use for lung or digestive cancers [2,4]. HCV is an oncogenic virus proven to cause hepatocarcinoma (HCC) and B-cell non-Hodgkin lymphoma [7,8]. Furthermore, some epidemiological studies suggest that patients with chronic HCV infection are also at higher risk than the general population of developing other cancers, such as cancers of the esophagus, pancreas, prostate, thyroid, breast or oral cavity [5,7,9–11]. In national surveys consecutively conducted in France in 2000, 2005 and in 2010, the rate of death attributed to non-AIDS-defining cancers and non HCV-liver (NANL) related cancers significantly increased between 2000 and 2010 (11% of deaths in 2000, 17% in 2005 and 22% in 2010, p<0.001) [12].

Evaluations of factors associated with NANL related cancers among HIV/HCV co-infected patients are scarce. In particular, the influence of HCV-related factors, such as duration of HCV infection, cirrhosis, HCV genotype and HCV viral load, needs further study. We aimed to investigate the associations between HIV/HCV clinical characteristics, and patients’ socio-behavioural profiles, and the occurrence of NANL related cancers in a large cohort of HIV/HCV-coinfected patients (ANRS CO13 HEPAVIH) followed in the era of increasing effectiveness of combination antiretroviral therapy (cART).

## Methods

This study involved HIV/HCV coinfected patients enrolled in the ANRS CO13 HEPAVIH cohort, a prospective, hospital-based cohort of HIV/HCV coinfected patients created in 2005 and involving 28 hospitals in France [13]. For this study, patients with at least one follow-up visit before 31^st^ September 2017 were included. Patients with a history of cancer at inclusion, patients with spontaneously cured HCV, those without follow-up, and those with missing data for tobacco consumption, alcohol consumption or cirrhotic status were excluded. The study was approved by the institutional review board: Comité Ile de France 3, file n°2234, ref CG/LG/CC 2005-255. Each participant agreed to participate to the ANRS CO13 Hepavih cohort by written consent.

The primary outcome was the time to occurrence of a first NANL related cancer. Cancer cases were prospectively collected. Patients with cancers were identified using the medical dictionary for regulatory activities (MedDRA). Cancer cases were also researched in the record deaths mentioning the cause of death. Patients were censored at the date of the diagnosis of a first NANL related cancer, at their date of death, or at their last follow-up, date whichever came first.

Qualitative variables were described as number (percentage) and quantitative variables as median (interquartile range [IQR]). Independent risk factors associated with NANL related cancer were studied using survival analysis. To take into account competitive risk for death without NANL related cancer, patients were censored at their death date if they didn’t experience a NANL cancer before.

First, univariable Cox proportional hazards models were used to identify factors associated with NANL related cancers. Variables included in these models were: age at inclusion (in years), sex, smoking status (never, former, current), alcohol consumption (never, former, current), time since HIV diagnosis (in years), duration of antiretroviral treatment (in years), AIDS status (yes, no), CD4 cell count nadir inferior to 200 cells/mm3 (yes, no), CD4 cell count (in cells/mm^3^), CD8 cell count (in cells/mm^3^), CD4/CD8 ratio, HIV viral load undetectable (yes, no), HCV transmission group (men who have sex with men, intravenous drug users, others), duration of HCV infection (counting from the date of the first transfusion, the date of initial intravenous drug use, or the first positive HCV serology in subjects who were infected via the sexual route and who did not develop acute hepatitis C), HCV genotype 1 (yes, no). Sustained viral response (SVR) to anti-HCV therapy (yes, no) defined as an undetectable serum HCV RNA (< 15 IU per milliliter) 24 weeks after treatment completion was included in the models as a time dependent variable. Cirrhosis status assessed by transient elastometry (TE) or FIB-4 score calculated as previously described [14] for patients without liver stiffness measurement. Cut-offs defining fibrosis were liver stiffness measurement superior or equal to 12.5 kPa and FIB-4 superior or equal to 3.45. Type 2 diabetes (yes, no) defined by a preexisting diagnosis of diabetes or an antidiabetic treatment at baseline, or a fasting blood glucose value of ≥7 mmol/L at least twice during follow-up. Wald’s tests were realized, and p-values were estimated.

Then a manual backward elimination procedure was realized in a Cox proportional hazards model. Variables with p-value inferior to 0.25 in univariate analysis were included in the model and factors *a priori* deemed to be important predictive factors of NANL cancers (age, sex, tobacco consumption, alcohol consumption, CD4 cell count and SVR) were forced in the model. Then variables not *a priori* important and with p-values superior to 0.05 were excluded from the model. The proportional hazards assumption was tested with interactions between covariates and time.

Cumulative incidences of NANL cancer were estimated for each quartile of time since HIV diagnosis using Aalen Johansen methodology to take into account competitive risk of death without NANL related cancer. All statistical tests were two-sided, with a type I error of 5%. Statistical analyses were performed using SAS version 9.4 (SAS Institute, Inc., Cary, North Carolina).

## Results

Among 1427 eligible patients, 36 were excluded due to missing data for tobacco-, alcohol-consumption or cirrhosis status. Therefore, a total of 1391 patients were considered for this analysis.

The demographic and clinical characteristics of the study population at baseline are shown in Table 1. Most patients were male (73%), and median age was 46 years (IQR, 43–51 years). There were respectively 69% current smokers and 49% current alcohol drinkers. The median time since HIV diagnosis was 18.6 years (IQR, 13.9–21.5). Most patients (90%) received cART, and the median duration of cART was 129 months (IQR, 82–179). AIDS was present in 25% of patients. The median CD4 cell count was 478 cells/mm^3^ (IQR, 325–675 cells/mm^3^), median CD4 cell count nadir was 155 cells/mm^3^ (IQR, 73–248 cells/mm^3^), and 75% of patients had an undetectable HIV viral load (<50 copies/ml). At baseline, 1173 patients (96%) had a positive HCV RNA and 27% had cirrhosis. The median duration of HCV infection was 14 years (IQR, 8.0–22).

**Table 1:** Baseline characteristics of the 1391 HIV/HCV-coinfected patients from the ANRS CO13 HEPAVIH cohort; results are medians (IQR) or numbers (%).

	Total (n = 1391)
Age (years)	46 (43–51)
Males	1009 (73)
**Smoking status**	
• Never	190 (13.6)
• Current	954 (68.6)
• Former	247 (17.8)
Alcohol consumption*	
• Never	306 (22)
• Current	682 (49)
• Former	403 (29)
**HIV infection**	
Time since HIV diagnosis (years)	18.6 (13.9–21.5)
AIDS	348 (25.3)
Ongoing antiretroviral treatment	1255 (90.4)
Duration of antiretroviral treatment (months) **	129.2 (82.4–178.7)
CD4 cell nadir (/mm^3^)	155 (73–248)
CD4 cell nadir <200(/mm^3^)	799 (62.6)
Absolute CD4 cell count (/mm^3^)	478 (325–675)
Absolute CD8 cell count (/mm^3^)	787 (546–1084)
CD4/CD8 ratio	0.6 (0.4–0.9)
HIV viral load < 50 copies/ml	1038 (74.8)
**HCV infection**	
HCV transmission group	
• MSM***	92 (7.6)
• *Intravenous drug users*	807 (66.8)
• *Others*	310 (25.6)
Duration of HCV infection (years)	14 (8–22)
HCV treatment-naïve patients	933 (67.1)
HCV genotype 1	750 (56.7)
Positive serum HCV RNA	1173 (96.2)
Cirrhosis	378 (27.2)
HCV viral load, log10 IU/mL	6.2 (5.6–6.6)
**Diabetes**	86 (6.2)
**HBs antigen positivity**	33 (2.4)

*>50 g/daily for men; >30 g/daily for women;

** zero in patients not receiving antiretroviral therapy;

***: Men who have sex with men

Median follow-up was 5 years (IQR, 1.9–7.5 years). Overall, 94 patients developed cancers, 7 AIDS defining cancers, 27 HCV related cancers and 60 NANL related cancers. Median time between cohort inclusion and cancer diagnosis was 2.7 years (IQR, 1.3–5.3). The incidence rate of NANL related cancers was 8.9 per 1000 person-years (95% CI, [6.6–11.1]). Median age at cancer diagnosis was 52 (IQR, 48–56) years. The types of these cancers are described in Table 2. The results of any search for HPV through histological reports were not available in our cohort. However, 9 patients (15%) had a high probability of HPV-related cancers (anal cancer n = 6, tonsil cancer n = 1, tongue cancer n = 1, penile cancer n = 1). Five patients (8.3%) had EBV related cancers (Hodgkin lymphoma in all). Non-melanoma skin cancers and lung cancers were the most frequent NANL related cancers with respectively 13 and 14 cases.

**Table 2:** The 60 non-AIDS-defining cancers and non HCV-liver related cancers diagnosed in HIV/HCV-coinfected patients.

Type of Cancer	Number of patients
Nasopharynx/Tongue/Tonsil cancer	6 (10)
Lung cancer	14 (23)
Anal cancer	6 (10)
Esophagus/colon/rectal/pancreas cancer	6 (10)
Breast or ovarian cancer	2 (3)
Hodgkin’s lymphoma	5 (8)
Non-melanoma skin cancer	13 (22)
Renal/urethra/prostate/penile cancer	5 (8)
Others	3 (5)

By univariable analysis, the following variables were related to the outcome with a p-value <0.25: age at baseline (HR = 1.09, 95% CI [1.05–1.15], p<0.0001) and duration since HIV diagnosis (HR = 1.06, 95% CI [1.01–1.13], p = 0.0281). Duration of cART, CD4 cell count nadir below 200/mm3 and duration of HCV infection tended to be associated with an increasing risk of NANL cancers while CD8 cells count tended to be associated with a decreasing risk. These factors were included in the multivariable analysis in addition to sex, tobacco, alcohol, CD4 cell count and SVR which forced in the model. Only age and a longer duration since HIV diagnosis remained independently associated with a higher risk for NANL related cancer (adjusted HR 1.10 for each additional year, 95% CI 1.06–1.14, p<0.0001 and adjusted HR 1.06 for each additional year, 95% CI 1.01–1.11, p = 0.02, respectively). SVR tended to decrease the risk of NANL related cancer but the association was not statistically significant (adjusted HR 0.71, 95% CI 0.21–2.32, p = 0.56) (Table 3).

**Table 3:** Factors associated with non-AIDS-defining cancers and non HCV-liver related cancers among HIV/HCV-coinfected patients—Univariate and multivariate analyses from the ANRS CO13 HEPAVIH Cohort (N = 1391).

	Univariable analysis		Multivariable analysis	
	RR [95% CI]	P	RR [95% CI]	P
Age	1.09 [1.05–1.14]	<0.0001	1.10 [1.06–1.14]	**<0.0001**
Female (versus Male)	0.84 [0.43–1.66]	0.61	0.79 [0.41–1.51]	0.43
Smoking status (versus never)		0.27		0.08
• Former	1.67 [0.42–6.68]		1.08 [0.34–3.45]	
• Current	2.40 [0.74–7.81]		2.22 [0.84–5.85]	
Alcohol consumption (versus never)		0.79		0.56
• Former	0.78[0.35–1.74]		0.66 [0.31–1.40]	
• Current	0.81 [0.40–1.64]		0.79 [0.41–1.51]	
Time since HIV diagnosis (years)	1.06 [1.01–1.13]	0.028	1.06 [1.01–1.11]	**0.026**
AIDS status	0.99 [0.50–1.96]	0.98		
Duration of ARV^1^ treatment (months)	1.00 [1.00–1.01]	0.066		
CD4 cell count nadir<200 cell/mm3)	1.62 [0.82–3.21]	0.16		
CD4 cell count (/mm^3^)^2^	1.00 [0.95–1.05]	0.98	0.98 [0.94–1.03]	0.46
CD8 cell count (/mm^3^) ^2^	0.98 [0.95–1.01]	0.19		
CD4/CD8 ratio	1.05 [0.53–2.10]	0.88		
Undetectable HIV RNA viral load	0.88 [0.47–1.65]	0.67		
HCV infection group (*vs* MSM)		0.65		
• Intravenous drug users	1.46 [0.35–6.08]			
• Others	1.09 [0.24–4.97]			
Duration of HCV infection (years)	1.03 [0.99–1.07]	0.13		
HCV genotype 1 versus others	0.97 [0.54–1.75]	0.91		
HCV sustained viral response (SVR)	0.48 [0.11–2.06]	0.32	0.71 [0.21–2.32]	0.56
Cirrhosis	0.99 [0.50–1.95]	0.97		
Diabetes	1.51 [0.54–4.21]	0.43		

^1^: antiretroviral treatment

^2^: for a difference of 50 cells/mm^3^

## Discussion

In this large prospective cohort of HIV/HCV-coinfected patients followed between 2005 and 2017, 60 patients developed NANL related cancers. Over this period of increasing effectiveness of cART, the overall incidence of NANL related cancers was 8.9 per 1000 person-years. In keeping with previous reports showing that the most important risk factors for non-AIDS-defining cancers were advancing age and the duration of HIV infection, age and the time since HIV diagnosis, after adjustment for other factors, were the only factors found to be independently associated with a higher risk of developing NANL related cancers in our study [2]. Conversely, despite a long history of chronic HCV infection in our patients (median 14 (8–22) years), no association was observed with HCV-related variables such as HCV transmission group, duration of HCV infection, cirrhosis, HCV genotype, HCV viral load or SVR.

Many questions regarding the relationship between HCV replication and non HCV-liver related cancers remain unanswered. It is well established that HCV replication is observed in both T and B lymphocyte subsets and is associated with chronic immune activation [15]. Large cohort studies have shown that chronic HCV infection is associated with an elevated risk of developing lung, pancreatic, oropharyngeal, thyroid, skin or breast cancers in HCV mono-infected patients [7,9–11,16]. HCV eradication following anti-HCV treatment has been associated with a high proportion of complete or partial response of B cell non-Hodgkin lymphoma both in HCV mono-infected patients and in HIV/HCV co-infected patients, supporting the role of chronic antigenic stimulation by HCV on lymphoma genesis [17,18]. Furthermore, HCV infection has also been associated with a higher risk of mortality from esophagus, prostate and thyroid cancers in HCV mono-infected patients with detectable HCV RNA, as compared to HCV mono-infected patients with undetectable HCV RNA [9]. In a retrospective Spanish study, HIV/HCV-coinfected patients had a higher cumulative incidence of NANL cancer than HIV-monoinfected patients (adjusted HR 1.26) [19]. However, in a study involving 1625 HIV/HCV-coinfected patients who were followed up for a median of five years after the end of treatment with interferon plus ribavirin, SVR was not associated with a reduced hazard of NANL related cancers [20]. In our study, patients with SVR tended to have a lower risk of NANL related cancer (aHR 0.71, 95% CI 0.21–2.32, p = 0.56) but the association was not statistically significant.

Impaired immunity is associated with a higher risk of EBV or HPV related cancer. In HIV mono-infected patients, the most frequent non-AIDS-related cancers are oncogenic virus associated malignancies such as EBV and HPV, and low CD4 counts have been associated with a higher risk of these cancers [2,6,21–23]. HCV infection has also been associated with a higher risk of anal cancer in non HIV patients [24]. Furthermore, a higher prevalence of high risk HPV has been observed among non-HIV female liver transplant candidates with HCV chronic infection compared to women without HCV infection [25]. The higher risk of HPV related cancers in HCV mono- infected patients could be related in part to impaired immunity due to cirrhosis. Indeed, cirrhosis has been associated with decreased monocyte function, altered natural killer activity and lectin-induced proliferation of T lymphocytes [25,26]. In our study, the role of HPV was probable in 15% of the cancers (anal cancer, tonsil cancer, tongue cancer and penile cancer). We found no association between a nadir CD4 cell count below 200/mm^3^, low current CD4 count or cirrhosis, and the occurrence of NANL related cancers. However, we could not evaluate the duration of CD4 count below 200/mm^3^ for most of our patients.

Surprisingly, tobacco or alcohol consumption, which are both well known risk factors for some cancers, such as oropharyngeal and lung cancers, were not found to be associated with the occurrence of NANL related cancers in our study, probably because these cancers were not specifically evaluated [27]. Low CD4/CD8 ratio has also been associated with non-AIDS defining cancer in patients on antiretroviral therapy but no such association was observed in our study [28].

The major strength of this large study (1391 patients followed in 28 hospitals throughout France) is that we adjusted for most factors (including socio-behavioral characteristics) potentially associated with NANL related cancers in patients with long duration of both HIV and HCV infection. The main limitation of our study is that despite a median follow-up of 5 years (IQR, 1.9–7.5 years), few cases of NANL related cancers occurred. Therefore, the non-significant results observed in our study, for example regarding the impact of CD4 cell count nadir, duration of HCV infection and SVR, could be related to a lack of power. Consequently, our results need to be confirmed in larger cohorts with longer follow-up.

In conclusion, age and the time since HIV diagnosis were the only factor found to be associated with an increased risk of NANL related cancers in this study. In contrast, no association was observed with any HCV-related variables. Since a marked increase in the rate of viral responses is observed in HIV/HCV coinfected patients receiving direct-acting anti-HCV drugs, further research is needed to determine whether suppression of HCV replication would have an impact on occurrence of some of these NANL related cancers.
